# A Risk Prediction Tool for Invasive Melanoma

**DOI:** 10.1001/jamadermatol.2025.3028

**Published:** 2025-09-10

**Authors:** David C. Whiteman, Catherine M. Olsen, Huanwei Wang, Matthew H. Law, Rachel E. Neale, Nirmala Pandeya

**Affiliations:** 1 Department of Population Health, QIMR Berghofer Medical Research Institute, Herston, Queensland, Australia; 2 School of Public Health, University of Queensland, Queensland, Australia; 3 School of Public Health, Queensland University of Technology, Queensland, Australia; 4 Frazer Institute, Faculty of Health, Medicine and Behavioural Sciences, The University of Queensland, Queensland, Australia; 5 School of Biomedical Sciences, University of Queensland, Queensland, Australia

## Abstract

**Importance:**

Increasingly, strategies to systematically detect melanomas invoke targeted approaches, whereby those at highest risk are prioritized for skin screening. Many tools exist to predict future melanoma risk, but most have limited accuracy and are potentially biased.

**Objectives:**

To develop an improved melanoma risk prediction tool for invasive melanoma.

**Design, Setting, and Participants:**

This population-based prospective cohort study (the QSkin Study) in Queensland, Australia, involved 10 years of follow-up from the baseline survey in 2011 and included individuals aged between 40 to 69 years who were melanoma-free at baseline and completed a comprehensive risk factor survey at recruitment. The data analysis was conducted from October 2024 to April 2025.

**Exposures:**

Thirty-one candidate variables collected at baseline were identified a priori as potential predictors of future risk of invasive melanoma.

**Main Outcomes and Measures:**

Histologically confirmed invasive melanomas newly diagnosed from baseline through to December 31, 2021, captured by data linkage to the Queensland Cancer Register. Follow-up was censored on diagnosis of melanoma in situ or death. Cox proportional hazards models with forward and backward selection approaches were used to identify the best-fitting model.

**Results:**

Of 41 919 eligible participants, 55% were female, and the mean (SD) age at baseline was 55.4 (8.2) years. A total of 706 new invasive melanomas were identified during 401 356 person-years of follow-up. The best-fitting model retained 14 predictors (age, sex, ancestry, nevus density, freckling density, hair color, tanning ability, adult sunburns, family history, other cancer prior to baseline, previous skin cancer excisions, previous actinic keratoses, smoking status, and height) and 2 statistical terms (age squared, age-by-sex interaction), yielding an apparent discriminatory accuracy of 0.74 (95% CI, 0.73-0.76). The Youden index was optimized at a screening threshold selecting the top 40% of predicted risk, which captured 74% of cases (number needed to screen = 32).

**Conclusions and Relevance:**

This cohort study has identified an improved tool that offers enhanced accuracy for predicting the future risk of invasive melanoma compared with existing tools.

## Key Points

**Question:**

Can a risk prediction tool for invasive melanoma be developed with better performance and less bias compared to existing prediction tools?

**Findings:**

In this cohort study of 41 919 participants with a 10-year follow-up, a new risk prediction tool for invasive melanoma was developed and, using 16 predictors, delivered improved prediction performances over previous tools. The new tool would identify 76% of future melanoma cases within 10 years by targeting individuals in the top 40% of predicted risk.

**Meaning:**

This prediction tool offers the potential for targeting individuals at higher risk of melanoma for systematic early detection activities.

## Teaser

This prospective cohort study with a 10-year follow-up period aims to develop and validate an improved melanoma risk prediction tool for invasive melanoma.

## Introduction

Melanomas are common and potentially fatal cancers arising in the pigment-producing cells of the skin. Survival from melanoma is correlated inversely with the thickness of the primary tumor.^1^ Because thin melanomas have a very low risk of mortality, efforts to control melanoma in many populations have focused on early detection, with the aim of diagnosing and completely excising tumors before deeper invasion and metastasis occur. For other common cancers (including cancers of the breast, cervix, prostate, and lung), screening programs have been established in many countries to successfully reduce mortality. However, population-based screening for melanoma has been implemented only in Germany; most health agencies internationally do not recommend routine melanoma screening for the general population based on insufficient evidence of overall benefit.^2^ That said, clinical guidelines and policies issued by leading organizations in the US, Australia, New Zealand, the UK, and the Netherlands recommend various models of targeted early detection based on risk stratification, in which persons identified as “high risk for melanoma or skin cancer” (variously defined) undergo periodic clinical skin examination.^3,4^

To assess individual risk, more than 40 melanoma prediction tools have been developed, including a tool we derived previously.^5^ That tool, comprising 7 predictors (melanoma predictor with 7 terms, hereafter, MP7), was the only melanoma prediction tool rated to have a low risk of bias by independent analysis.^6^ While the MP7 tool had good discrimination (C-index, 0.69 [95% CI, 0.62-0.76] in the validation dataset) and reasonable calibration, it was developed from a relatively small number of melanoma events using now-outdated approaches. Since that time, statistical methods for predicting risk have evolved considerably, including new approaches to harness all available data, build models, and evaluate performance.^7,8,9^ Moreover, the number of newly diagnosed invasive melanomas in the QSkin cohort has almost tripled, providing substantially greater statistical power than previously. Herein, we describe the development and validation of a new prediction model for invasive melanoma in the QSkin cohort over 10 years to inform risk-stratification strategies.

## Methods

### Study Population

We analyzed data from the QSkin Sun and Health Study, methodological details of which have been published.^10^ Briefly, QSkin is a prospective cohort study of a large group of adults aged 40 to 69 years sampled randomly from the Queensland, Australia, population from November 2010 to December 2011. Participants with a registry-confirmed diagnosis of melanoma (in situ or invasive) before baseline were excluded. The Human Research Ethics Committee at the QIMR Berghofer Medical Research Institute approved the study (P1309, P2034), and all QSkin participants gave their written informed consent to take part. We followed the Transparent Reporting of a Multivariable Prediction Model for Individual Prognosis or Diagnosis (TRIPOD) reporting guideline for model development and performance assessment.

### Data Collection

At baseline, participants provided detailed melanoma risk factor data using a self-completed questionnaire with high to very high repeatability for most items.^11,12^ The baseline survey instrument is freely available online.^13^ All participants consented for their records to be linked to the Queensland Cancer Register (notification of histologically confirmed melanoma is mandatory).

### Candidate Predictor Variables

A prespecified list of 31 predictor variables (comprising 27 phenotypic/clinical variables and 4 statistical interaction variables) was derived based on previously identified risk factors, as well as potential predictors of clinical significance from the literature (eMethods, eTable 1 in Supplement 1).

### Imputation of Missing Data

Missing value prevalence was generally low (<3%) but ranged up to 15% for family history of melanoma (eTable 1 in Supplement 1). To minimize potential bias due to missing data, multivariate imputation by chained equations was performed, assuming data were missing completely at random, and created 50 multiply imputed datasets (eMethods in Supplement 1).

### Main Outcomes

The primary outcome was first histological diagnosis of invasive melanoma ascertained through linkage to the Queensland Cancer Register. We did not include in situ melanomas as outcomes of clinical interest in prediction models, given widespread concerns about overdiagnosis of such lesions. Instead, histologically confirmed diagnoses of in situ melanoma were recorded and handled through censoring.

### Statistical Analysis

We used Cox proportional hazards approaches to derive a risk prediction model for the onset of newly diagnosed invasive melanoma. We used all available data to develop the model and used resampling methods for internal validation.

Follow-up time commenced at the date of study entry and continued until a maximum of 10 years duration. Follow-up time ceased on the date of the first histologically confirmed diagnosis of invasive cutaneous melanoma, or at 10 years follow-up, whichever occurred first. We censored participants early on the date of their death or the date of diagnosis of in situ melanoma, if those events occurred before the primary outcome or administrative censoring date.

We applied a stepwise approach to variable selection using the full set of potential predictors. We used the minimum Akaike information criterion (AIC) method and included both forward selection and backward elimination processes. We executed variable selection for each of the 50 imputed datasets. Predictors selected in at least 35 of 50 imputed datasets (70%) were retained in the final risk prediction model. To estimate the apparent effect estimates, we pooled effect estimates from the final model from each of the 50 imputed datasets using Rubin rules.^14^ Cumulative hazards were log transformed before pooling, and the pooled value was then exponentiated. We assessed the proportional hazard assumptions for the predictors included in the final developed model in all 50 imputed datasets individually. Analyses were performed in SAS statistical software, version 9.4 (SAS Institute Inc) and R statistical software, version 4.4.2 (R Project for Statistical Computing) (eMethods in Supplement 1). The data analysis was conducted from October 2024 to April 2025. Two-sided *P* values less than .05 were deemed statistically significant.

### Model Performance and Internal Validation

We assessed model performance using discrimination and calibration. We calculated the concordance index (C-index) and Brier score (mean squared error of predictions) for the final developed risk prediction model in each imputed dataset and reported the pooled estimates of these performance metrics.

For internal validation and calibration, we generated 1000 bootstrap samples from one of the multiply imputed datasets. We used the full list of candidate predictors and stepwise selection with minimum AIC for validation and calibration, and used bootstrap samples to estimate the optimism-corrected index, as recommended (eMethods in Supplement 1).^15^

To assess the stability of effect estimates, we plotted estimated risk at 10 years based on the final developed model using one imputed dataset against the same estimates from the 1000 bootstrap sample models. We estimated the mean absolute prediction error for each individual as an average of the absolute difference between the risk predicted by the developed risk prediction model and each of the 1000 bootstrap models.

### Clinical Utility

We grouped participants by deciles of predicted risk and estimated their cumulative hazard at 10 years. We calculated the Youden index for each decile to assess the threshold at which both sensitivity and specificity were optimized.^16^ We performed decision curve analysis to assess the net benefit of using the model for screening at different thresholds compared to screening all and screening none.^17^

Lastly, we compared the performance of the new tool against the MP7 tool^5^ by comparing sensitivities of the 2 models at decile cut points to 10 years of follow-up, and then calculating the net reclassification index (NRI) under different thresholds for targeted screening.^18^

### Sensitivity Analysis

We conducted 2 sensitivity analyses: (1) we assessed the performance of the derived model in the QSkin dataset, which ignored all diagnoses of melanoma in situ; and (2) we separately rederived models ignoring all diagnoses of melanoma in situ.

## Results

### Participants

**Figure 1.**
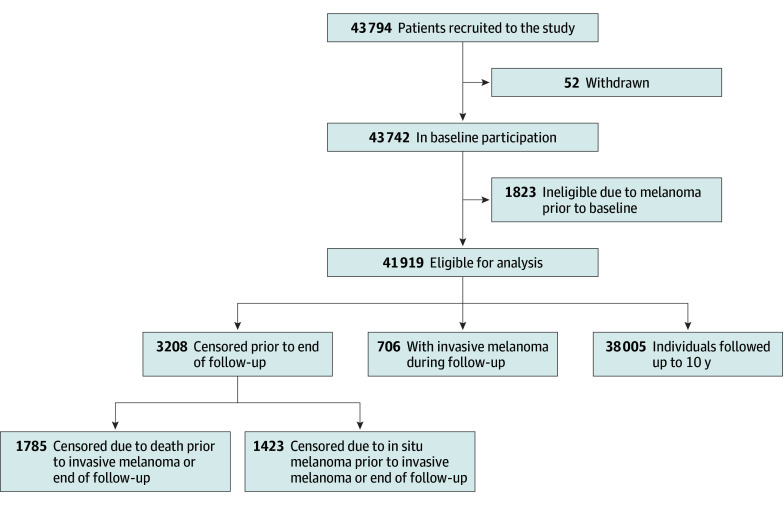
Flowchart for Participant Recruitment and Outcomes in the QSkin Study of Sun and Health

Among 43 794 participants, 1823 had a registry-confirmed melanoma diagnosis (in situ or invasive) prior to baseline and were excluded, and 52 withdrew subsequently, leaving 41 919 eligible participants for this analysis (Figure 1). Of 41 919 eligible participants, 22 942 (55%) were female, and the mean (SD) age at baseline was 55.4 (8.2) years. Most participants (n = 38 120 [94%]) reported having European ancestry, which is considered high risk for developing melanoma. Distributions of factors retained in the final model are provided in Table 1 by outcome status; other candidate predictor variables are presented in eTable 1 in Supplement 1. During follow-up (401 356 person-years; mean [SD], 9.6 [1.6] years; median 10.0 [IQR, 10.0-10.0] years), 706 participants (1.7%) developed an invasive melanoma (mean [SD], 5.0 [2.9] years; median 5.1 [IQR, 2.4-7.4] years); the clinical characteristics of the melanomas are provided in eTable 2 in Supplement 1.

**Table 1.** Demographic, Socioeconomic, and Phenotypic Characteristics Retained in the Final Developed Model

Characteristic	No. (%)			*P* value^a^
	Overall (N = 41 919)	No melanoma (n = 41 213)	Invasive melanoma (n = 706)	
Age at baseline, mean (SD), y	55.4 (8.2)	55.4 (8.2)	57.9 (7.5)	<.001
Height, mean (SD), cm				
Female	163.7 (7.0)	163.7 (7.0)	164.2 (6.3)	.14
Male	177.3 (7.1)	177.3 (7.1)	178.0 (7.6)	.04
Missing, No.	901	893	8	
Sex				
Female	22 942 (55)	22 633 (55)	309 (44)	<.001
Male	18 977 (45)	18 580 (45)	397 (56)	
Ancestral risk of melanoma				
Low/moderate risk	2625 (6.3)	2612 (6.4)	7 (1)	<.001
High risk	38 930 (94)	38 120 (94)	696 (99)	
Missing, No.	364	361	3	
Nevus density at 21 y of age				
None	11 769 (29)	11 615 (29)	154 (22)	<.001
A few	21 751 (53)	21 417 (53)	334 (48)	
Some	6033 (15)	5869 (15)	164 (24)	
Many	1231 (3)	1191 (3)	40 (6)	
Missing, No.	1135	1121	14	
Hair color				
Black	4238 (10)	4200 (10)	38 (5.4)	<.001
Dark/light brown/blonde	35 130 (84)	34 546 (84)	584 (83)	
Red/auburn	2297 (6)	2218 (5)	79 (11)	
Missing, No.	254	249	5	
Freckles at 21 y of age				
None	19 816 (48)	19 577 (48)	239 (34)	<.001
A few	12 984 (31)	12 754 (31)	230 (34)	
Some	6396 (15)	6224 (15)	172 (25)	
Many	2472 (6)	2411 (6)	61 (9)	
Missing, No.	251	247	4	
Tanning ability				
Tan deeply	9807 (24)	9717 (24)	90 (13)	<.001
Tan moderately	20 527 (49)	20 202 (49)	325 (47)	
Tan lightly	8622 (21)	8425 (21)	197 (28)	
Not tan	2623 (6)	2537 (6)	86 (12)	
Missing, No.	340	332	8	
Sunburns as an adult				
None	6718 (17)	6637 (17)	81 (12)	<.001
1-10	27 696 (69)	27 207 (69)	489 (71)	
11-20	3516 (9)	3454 (9)	62 (9)	
>20	2034 (5)	1982 (5)	52 (8)	
Missing, No.	1955	1933	22	
Family history of melanoma				
No	26 226 (74)	25 816 (74)	410 (67)	<.001
Yes	9315 (26)	9109 (26)	206 (33)	
Missing, No.	6378	6288	90	
Any other cancer prior to baseline				
No	38471 (92)	37861 (92)	610 (86)	<.001
Yes	3448 (8)	3352 (8)	96 (14)	
Baseline smoking status				
Never	22 816 (55)	22 400 (55)	416 (59)	.02
Past	14 869 (36)	14 633 (36)	236 (34)	
Current	4057 (10)	4005 (10)	52 (7)	
Missing, No.	177	175	2	
Skin cancer excisions prior to baseline				
None	26 069 (63)	25 800 (63)	269 (39)	<.001
1	5596 (13)	5493 (13)	103 (15)	
>1	9940 (24)	9614 (24)	326 (47)	
Missing, No.	314	306	8	
Sunspots treated prior to baseline				
None	19 531 (47)	19 374 (47)	157 (22)	<.001
1-5	10 948 (26)	10 751 (26)	197 (28)	
6-20	6903 (17)	6726 (16)	177 (25)	
>20	4301 (10)	4128 (10)	173 (25)	
Missing, No.	236	234	2	

^a^ Pearson χ^2^ test was used for categories, and Wilcoxon rank sum test was used for continuous predictors.

Participants with invasive melanoma were older (mean [SD] age, 57.9 [7.5] years vs 55.4 [8.2] years; *P* < .001), more likely to be male (397 of 706 [56%] vs 18 580 of 41 213 [45%]; *P* < .001), born in Australia (605 of 706 [86%] vs 32 909 of 41 213 [80%]; *P* < .001), and report European ancestry (696 of 706 [99%] vs 38 234 of 41 213 [94%]; *P* < .001) than those who remained free of melanoma. They were also more likely to have fair pigmentation (red or light hair and blue eye color) and a higher self-reported nevus density (Table 1; eTable 1 in Supplement 1).

At the administrative end date, 91% of participants were event free. Among 3208 censored participants, 1423 (44%) were censored due to a diagnosis of melanoma in situ, and 1786 (56%) due to death. Censoring events occurred uniformly over the follow-up period (eFigure 1 in Supplement 1).

### Model Specification

Sixteen terms were retained in the final model (hereafter, MP16), comprising 14 clinical predictors and 2 statistical terms (age squared, age-by-sex interaction; Table 2). Factors associated with the highest risks of incident invasive melanoma were European ancestry, high nevus count, red hair color, and inability to tan. There was very high concordance across the 50 imputed datasets for the variables retained in the final prediction model (eTable 3 in Supplement 1). The prediction model met the proportional hazards assumption for all predictors except for the age-squared term (eTable 4 in Supplement 1); however, the effect estimate for age squared did not demonstrate marked nonproportionality over time and so was retained.

**Table 2.** Specification and Performance of the Developed Model to Predict Risk of Invasive Melanoma: Pooled Effect Estimates Applied to 50 Multiply Imputed Datasets

Predictor	Apparent effect estimates		
	β (SE)	HR (95% CI)	*P* value
Age^a^	0.02 (0.01)	1.02 (1.01 to 1.04)	.003
Age squared	−0.001 (0.001)	0.99 (0.99 to 1.00)	.02
Sex			
Female	0 [Reference]	1 [Reference]	NA
Male	0.49 (0.08)	1.63 (1.38 to 1.92)	<.001
Ancestral risk of melanoma			
Moderate/low risk	0 [Reference]	1 [Reference]	NA
High risk	1.16 (0.38)	3.20 (1.51 to 6.79)	.002
Nevus density at 21 y of age			
None	0 [Reference]	1 [Reference]	NA
A few	0.31 (0.10)	1.36 (1.12 to 1.66)	.002
Some	0.84 (0.12)	2.32 (1.85 to 2.91)	<.001
Many	1.10 (0.18)	3.01 (2.10 to 4.31)	<.001
Hair color			
Black	0 [Reference]	1 [Reference]	NA
Dark/light brown/blonde	0.46 (0.17)	1.58 (1.13 to 2.21)	.01
Red/auburn	0.77 (0.21)	2.15 (1.43 to 3.25)	<.001
Freckles at 21 y of age			
None	0 [Reference]	1 [Reference]	NA
A few	0.20 (0.10)	1.22 (1.01 to 1.47)	.04
Some	0.44 (0.11)	1.55 (1.25 to 1.92)	<.001
Many	0.24 (0.16)	1.27 (0.93 to 1.74)	.14
Tanning ability			
Tan deeply	0 [Reference]	1 [Reference]	NA
Tan moderately	0.31 (0.12)	1.37 (1.08 to 1.73)	.01
Tan lightly	0.53 (0.13)	1.69 (1.30 to 2.20)	<.001
Not tan	0.74 (0.17)	2.11 (1.52 to 2.91)	<.001
Sunburns as an adult			
None	0 [Reference]	1 [Reference]	NA
1-10	0.31 (0.12)	1.36 (1.07 to 1.73)	.01
11-20	0.15 (0.17)	1.17 (0.83 to 1.63)	.37
>20	0.48 (0.18)	1.62 (1.14 to 2.31)	.01
Family history of melanoma			
No	0 [Reference]	1 [Reference]	NA
Yes	0.15 (0.09)	1.16 (0.97 to 1.38)	.10
Other cancer prior to baseline			
No	0 [Reference]	1 [Reference]	NA
Yes	0.47 (0.11)	1.61 (1.29 to 2.00)	<.001
Baseline smoking status			
Never smoker	0 [Reference]	1 [Reference]	NA
Past smoker	−0.22 (0.08)	0.80 (0.68 to 0.94)	.01
Current smoker	−0.23 (0.15)	0.80 (0.60 to 1.07)	.12
Skin cancer excisions prior to baseline			
None	0 [Reference]	1 [Reference]	NA
1	0.21 (0.12)	1.24 (0.98 to 1.57)	.07
>1	0.44 (0.10)	1.55 (1.27 to 1.89)	<.001
Sunspots treated prior to baseline			
None	0 [Reference]	1 [Reference]	NA
1-5	0.46 (0.11)	1.59 (1.27 to 1.98)	<.001
6-20	0.50 (0.13)	1.65 (1.29 to 2.11)	<.001
>20	0.66 (0.14)	1.94 (1.47 to 2.54)	<.001
Height (per change in 1 unit standardized score)^b^	0.09 (0.04)	1.09 (1.01 to 1.18)	.02
Age-by-sex interaction	0.03 (0.01)	1.03 (1.01 to 1.05)	.01

Abbreviations: HR, hazard ratio; NA, not applicable; SE, standard error.

^a^ For age, the mean-corrected value was used in the Cox proportional hazards model.

^b^ For height, the sex-specific standardized score was used in the Cox proportional hazards model (eMethods in Supplement 1).

### Model Performance

**Figure 2.**
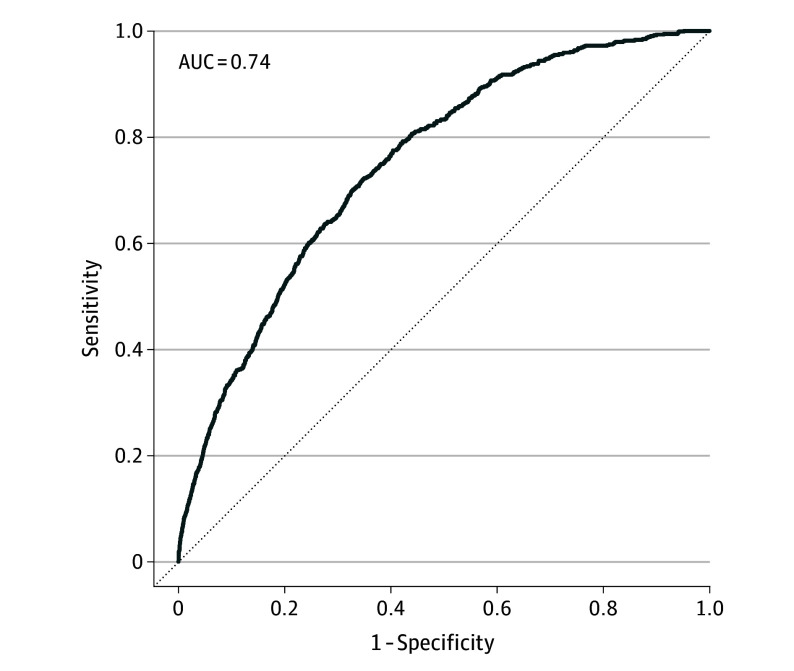
Area Under the Curve (AUC) in Single-Imputed Dataset for the Developed Model

The baseline cumulative hazard was 0.00050 at 10 years. The apparent pooled C-index of the model was 0.74 (95% CI, 0.73-0.76; Figure 2); this estimate was stable over time from approximately 1.5 years of follow-up (eFigure 2 in Supplement 1). The apparent pooled mean Brier score was 0.017, and *R^2^* was 0.039. The model showed better calibration between observed and predicted risk at lower risk levels than at higher levels, where it tended to overestimate risk (eFigure 3 in Supplement 1).

### Internal Validation and Calibration

The model selection process was run again in 1000 bootstrap samples, generating prediction models with a range of 6 to 19 factors (eTable 5 in Supplement 1). The C-index from bootstrap internal validation was 0.73 (95% CI, 0.71-0.76; eFigure 4 in Supplement 1). Internal validation estimated a uniform shrinkage of 0.929, yielding an optimism-corrected C-index of 0.71; the optimism-corrected Brier score was 0.016, and *R^2^* was 0.033.

The parameterwise joint shrinkage for most predictors in the developed model was 0.90 or greater, suggesting little bias due to overfitting (eTable 6 in Supplement 1). The global shrinkage estimate based on the DFBetas residuals (ie, difference in betas for each observation when that observation is removed from the analysis) was 0.941.

### Model Stability

The mean absolute prediction error between the apparent model and bootstrap models was 0.0046 (range, 0.0001-0.0818); the prediction error was greatest in the small proportion of individuals with very high predicted risks (eFigure 5 in Supplement 1).

### Clinical Utility

The Youden index was optimized with a value of 35 at the seventh decile (ie, the top 40% of predicted risk in the general population; Table 3). At this threshold, a targeted screening program would potentially capture 74% of all invasive melanoma cases in the population with a number needed to screen of 32. Reducing the risk threshold by 1 decile (ie, expanding the target to include people at the top 50% of predicted risk) would potentially capture 82% of invasive melanoma cases, with a number needed to screen of 36. Decision curve analysis showed that using the prediction model delivered a net benefit compared with screening all or screening none across a plausible range of threshold probabilities for 10-year risk (eFigure 6 in Supplement 1).

**Table 3.** Performance of Melanoma Screening Within Deciles of Predicted Risk Score for Invasive Melanoma

Predicted risk score decile	Sensitivity, %	Specificity, %	Youden index	Cumulative No. needed to screen
10th	32.6	90.4	0.23	18
9th	48.4	80.5	0.29	25
8th	63.6	70.6	0.34	28
7th	74.4	60.6	0.35	32
6th	82.2	50.6	0.33	36
5th	89.7	40.5	0.30	40
4th	94.3	30.4	0.25	44
3rd	97.2	20.3	0.17	49
2nd	99.0	10.2	0.09	54
1st	100.0	0	0.00	59

### Comparison With Previous Model

Compared with the performance of the previously derived MP7 model, the newly derived MP16 model predicted more true cases (n = 580) than the MP7 model (n = 545) down to the sixth decile, resulting in improvements to the performance of a targeted screening threshold at both the top 40% (NRI, 0.048) and top 50% (NRI, 0.050; eTable 7 in Supplement 1).

### Sensitivity Analysis

Changes in performance were negligible when comparing outputs from datasets that did and did not censor melanoma in situ diagnoses (eTable 8 in Supplement 1). Second, the model derivation steps were repeated in the imputed datasets by forcing the model to ignore melanomas in situ. The process resulted in a model with the same list of predictor variables as the MP16 model, and the inclusion of 1 new predictor (sunbed use); the hazard ratios of the retained predictors were very similar to those in MP16 (eTable 8 in Supplement 1).

## Discussion

Risk-stratified approaches appear to offer the most promising strategy for detecting melanomas efficiently at the level of the population. In theory, these approaches seek to identify those members of the population likely to experience the highest burden of melanoma and offer them skin examinations, while obviating low-value screening of people at lesser risk. More than 40 tools have been developed previously to predict an individual’s risk of melanoma, almost all of which have been assessed independently to be at high risk of bias. The only tool to be rated as low risk of bias was the 2018 QSkin tool (MP7).^6^ In this cohort study with 10 years of follow-up, we have improved on that earlier model by optimizing it with a longer follow-up time, more individuals with invasive melanoma, and new approaches to derive and validate the prediction model.

The new MP16 tool identified 14 independent risk factors and 2 statistical terms, which, when combined, delivered good discriminatory accuracy (C-index, 0.74). The model retained all 6 of the independent factors identified in MP7 (noting that in the new analysis, we did not consider “sunscreen use when outdoors in the past year” as a potential predictor, due to its lower repeatability and discretionary nature). The fit was improved by predictors newly retained in the MP16 model that captured ancestry, clinical factors (personal history of cancer, family melanoma history, past history of skin cancer excisions), sun exposure (number of sunburns as an adult), and phenotype (freckling density at 21 years of age). In addition, the fit of the model was improved significantly by including terms for both smoking and height; past and current smoking were associated with lower risks of melanoma (as reported consistently in the literature)^19^; and sex-standardized height (eMethods in Supplement 1) was positively associated with higher risks of melanoma. The final, best-fitting MP16 model also included terms for age-squared effects and age-by-sex interactions, capturing nonlinear patterns within the data. We observed almost uniform consistency in the frequency with which these 16 statistical terms were retained when we reran the derivation procedure separately within 50 imputed datasets. Similarly, these 16 terms were also those retained most frequently during validation.

We note that a number of promising candidate predictors were not retained during the process of deriving the model, including skin burning tendency, eye color, number of sunburns as a child or youth, ever use of sunbeds, and age at migration to Australia. Each of these factors has been identified in other studies as increasing the risk of melanoma. However, none of these terms was selected in more than 1% of 1000 bootstrapped models, indicating that their explanatory power was negligible when other factors were already included. The fact that such factors were not retained in the final model should not be inferred as evidence that these factors have no causal role in melanoma. Testing for causality must follow a different statistical approach, accounting for mediation, confounding, and direction of effect.

We emphasize that the primary end point in our analyses was first invasive melanoma, principally because, especially in Australia but also in the US, there is strong evidence of widespread overdiagnosis of melanomas in situ. We contend that there is no clinical utility in increasing efforts toward encouraging even more diagnoses of such lesions, which would likely serve only to exacerbate the problem of overdiagnosis. Moreover, because there is considerable debate about the biological characteristics of melanomas in situ (specifically whether or not they are all destined to invade),^20^ and because the natural history of such lesions is altered irreversibly by their excision, we censored people diagnosed with preinvasive melanomas in our primary analysis. In sensitivity analyses, we ignored melanomas in situ and followed up only for individuals’ first occurrence of invasive melanoma; we found that the same terms were identified, indicating that the final form of this prediction model does not depend on how the model was technically specified.

There is emerging consensus that a systematic approach to the early detection of melanoma will involve some form of targeted screening among those identified as at high risk for melanoma.^2^ For example, the American Cancer Society suggests that people with a family history of melanoma undergo regular skin examination by a dermatologist.^21^ Cancer Research UK advises that people with a strong family history of melanoma, or who have had more than 1 melanoma previously, are at higher than average risk and may be advised to have regular appointments with a dermatologist to check their skin.^22^ The clinical guidelines for melanoma issued by Cancer Council Australia in 2021 recommend that all patients should be assessed for “future risk of melanoma, using validated risk factors and a model that integrates personal risk factors into an overall index of risk.”^23^ Similarly, the Royal Australian College of General Practitioners (representing primary care physicians who diagnose and excise approximately 80% of primary melanomas in Queensland, Australia^24^) recommends using validated melanoma risk assessment tools to determine risk level, and that individuals at high risk of developing melanoma or keratinocyte cancer should undergo opportunistic examination of the skin.^25^ In 2024, the Australian government announced plans to investigate the feasibility of a national, targeted skin cancer screening program.^26^ That initiative aims to develop risk-based screening approaches to stratify the population and optimize screening efforts.

### Strengths and Limitations

A limitation of this analysis was our inability to validate the new prediction tool in an independent sample. This reflects the absence globally of established cohorts that have recruited both males and females and also collected comprehensive melanoma risk factor data at baseline. To address this limitation, we evaluated all aspects of model derivation and performance using bootstrapping.^9^ The QSkin MP16 prediction model has notable strengths. The cohort was sampled at random from the general population, and the prospective design ensures that baseline reporting of risk factors was not influenced by melanoma status. Follow-up was long (10 years), and histologically confirmed cases of invasive melanoma were identified through the cancer registry with essentially universal registration. The analytic approach and reporting followed TRIPOD principles, included prespecification of a defined list of candidate predictor variables, imputation for missing data, and extensive evaluation using bootstrap resampling to assess model stability and reduce overfitting.^7,8,9,15^

The new MP16 tool, incorporating factors captured easily by self-report, provides higher discriminatory accuracy than any previous tools, is well calibrated to 10 years, and has high internal validity. At the population level, we estimate that more than 80% of people who subsequently develop melanomas would be identified by this tool as higher-than-median risk for melanoma, at the cost of screening approximately 36 people who would not go on to develop melanoma, suggesting a pragmatic threshold for testing the feasibility of targeted screening at scale. How such tools are implemented in practice, from which age, and using which risk threshold to triage screening, are questions that remain to be resolved.

## Conclusions

This cohort study has identified an improved tool that offers enhanced accuracy for predicting the future risk of invasive melanoma compared with existing tools. In future research, we will assess the benefit of adding genetic information to the tool, and we have recently recruited a second, independent cohort in which to validate the tool externally in the years ahead. In the meantime, we see strong merit in assessing the performance of this tool independently in other settings.

## Notes

Supplement 1. **eMethods.** Supplementary methods **eTable 1.** Distribution of demographic, socio-economic and phenotypic characteristics that were not retained in the final model: overall for the QSkin cohort (n=41,919) and according to incident invasive melanoma status **eTable 2.** Tumour characteristics of the invasive melanomas included in the risk prediction model (n=706) **eTable 3.** Frequency of selection of candidate predictors from 50 imputed datasets during model derivation using minimum AIC selection criteria **eTable 4.** Assessment of the proportional hazards assumption for the 16 predictors retained in the final model **eTable 5.** Frequency of selection of candidate predictors during model selection in 1000 bootstrapped datasets, in descending order **eTable 6.** Parameter-wise shrinkage and bias-corrected beta estimates (pooled estimates of 50 multiply imputed datasets) **eTable 7.** Case distribution in risk deciles based on the previous QSkin risk prediction model and the current model for invasive melanoma after 10 years follow-up **eTable 8.** Results of sensitivity analyses ignoring incident melanoma in situ (pooled estimates of 50 multiply imputed datasets) **eFigure 1.** Survival probability for the outcome (invasive melanoma) and censoring events (melanoma in situ, death) over 10 years of follow-up **eFigure 2.** Time dependent C-index* for the developed model. (*single imputed dataset) **eFigure 3.** Observed versus predicted risk for (a) the developed model; and (b) the 1000 bootstrap sample (single imputed dataset) **eFigure 4.** Area under the curve for the 1000 bootstrap sample (single imputed dataset) **eFigure 5.** Mean absolute prediction error and estimated risk at 10 years using final risk prediction model for each individual **eFigure 6.** Decision curve analysis plotting net benefit against the threshold probability

Supplement 2. Data sharing statement
