# Supplementary material for: A Risk Prediction Tool for Invasive Melanoma
Source: JAMA Dermatol. 2025 Sep 10;161(11):1123–31. doi: 10.1001/jamadermatol.2025.3028 (PMC12423951; doi:10.1001/jamadermatol.2025.3028)
Supplement: Supplement 1. — eMethods. Supplementary methods eTable 1. Distribution of demographic, socio-economic and phenotypic characteristics that were not retained in the final model: overall for the QSkin cohort (n=41,919) and according to incident invasive melanoma status eTable 2. Tumour characteristics of the invasive melanomas included in the risk prediction model (n=706) eTable 3. Frequency of selection of candidate predictors from 50 imputed datasets during model derivation using minimum AIC selection criteria eTable 4. Assessment of the proportional hazards assumption for the 16 predictors retained in the final model eTable 5. Frequency of selection of candidate predictors during model selection in 1000 bootstrapped datasets, in descending order eTable 6. Parameter-wise shrinkage and bias-corrected beta estimates (pooled estimates of 50 multiply imputed datasets) eTable 7. Case distribution in risk deciles based on the previous QSkin risk prediction model and the current model for invasive melanoma after 10 years follow-up eTable 8. Results of sensitivity analyses ignoring incident melanoma in situ (pooled estimates of 50 multiply imputed datasets) eFigure 1. Survival probability for the outcome (invasive melanoma) and censoring events (melanoma in situ, death) over 10 years of follow-up eFigure 2. Time dependent C-index* for the developed model. (*single imputed dataset) eFigure 3. Observed versus predicted risk for (a) the developed model; and (b) the 1000 bootstrap sample (single imputed dataset) eFigure 4. Area under the curve for the 1000 bootstrap sample (single imputed dataset) eFigure 5. Mean absolute prediction error and estimated risk at 10 years using final risk prediction model for each individual eFigure 6. Decision curve analysis plotting net benefit against the threshold probability [file jamadermatol-e253028-s001.pdf]

Supplemental Online Content

Whiteman DC, Olsen CM, Wang H, Law MH, Neale RE, Pandeya N. Validity of risk prediction tool for invasive melanoma. *JAMA Dermatol.* Published online September 10, 2025. doi:10.1001/jamadermatol.2025.3028

eMethods. Supplementary methods

eTable 1. Distribution of demographic, socio-economic and phenotypic characteristics that were not retained in the final model: overall for the QSkin cohort (n=41,919) and according to incident invasive melanoma status

eTable 2. Tumour characteristics of the invasive melanomas included in the risk prediction model (n=706)

eTable 3. Frequency of selection of candidate predictors from 50 imputed datasets during model derivation using minimum AIC selection criteria

eTable 4. Assessment of the proportional hazards assumption for the 16 predictors retained in the final model

eTable 5. Frequency of selection of candidate predictors during model selection in 1000 bootstrapped datasets, in descending order

eTable 6. Parameter-wise shrinkage and bias-corrected beta estimates (pooled estimates of 50 multiply imputed datasets)

eTable 7. Case distribution in risk deciles based on the previous QSkin risk prediction model and the current model for invasive melanoma after 10 years follow-up

eTable 8. Results of sensitivity analyses ignoring incident melanoma in situ (pooled estimates of 50 multiply imputed datasets)

eFigure 1. Survival probability for the outcome (invasive melanoma) and censoring events (melanoma in situ, death) over 10 years of follow-up

eFigure 2. Time dependent C-index* for the developed model. (*single imputed dataset)

eFigure 3. Observed versus predicted risk for (a) the developed model; and (b) the 1000 bootstrap sample (single imputed dataset)

eFigure 4. Area under the curve for the 1000 bootstrap sample (single imputed dataset)

eFigure 5. Mean absolute prediction error and estimated risk at 10 years using final risk prediction model for each individual

© 2025 Whiteman DC et al. *JAMA Dermatology*.

eFigure 6. Decision curve analysis plotting net benefit against the threshold probability

This supplemental material has been provided by the authors to give readers additional information about their work.

© 2025 Whiteman DC et al. *JAMA Dermatology*.

eMethods.

Supplementary methods for Whiteman et al. "Risk of new-onset invasive melanoma to 10 years in a prospective cohort: a new melanoma risk prediction tool."

Study population

We analysed data from the QSkin Sun and Health Study, methodologic details of which have been published.[1] Briefly, QSkin is a prospective cohort study of 43,794 men and women aged 40-69 years sampled randomly from the Queensland population in 2010-2011. Among all participants, 1,823 had a registry-confirmed melanoma diagnosis (*in situ* or invasive) prior to baseline and were excluded, and 52 withdrew subsequently, leaving 41,919 eligible participants for this analysis (Figure 1).

Data Collection

At baseline, participants provided detailed data on demographic and phenotypic variables, sun exposure, general medical history, and skin cancer history using a self-completed questionnaire with high to very high repeatability for most items.[2,3] The baseline survey instrument is freely available [https://www.qimrb.edu.au/studies/qskin]. All participants consented for their records to be linked to the Queensland Cancer Register (QCR) to ascertain confirmed melanoma diagnoses for eligible participants up to December 31, 2021 (notification is mandatory).

Candidate Predictor Variables

From the comprehensive survey dataset, we pre-specified a list of 31 predictor variables (comprising 27 phenotypic/clinical variables and four statistical interaction variables) based on previously identified risk factors and their correlates, as well as potential predictors of clinical significance from the literature. The list of candidate predictors was approved by all authors prior to analysis. The candidate predictors for the full model were age at baseline, squared term for age at baseline, sex, education, work status, socio-economic disadvantage score, remoteness of area of residence, age at migration to Australia, primary place of residence before age 20 years, ancestral melanoma risk (derived from ancestry questions), nevus density and freckling density at age 21 years, hair colour, eye colour, skin burning tendency, skin tanning ability, sunburns as a child, sunburns as an adolescent, sunburns as an adult, tanning bed use, family history of melanoma, personal history of other cancer, smoking status, past history of skin cancer excisions (not melanomas), history of non-surgical treatment for skin lesions, alcohol consumption, and height. The four pre-selected interaction terms were age at baseline by sex, age by nevus density at 21, age by history of skin cancer excisions, and age by history of non-surgical treatment for skin lesions. Age, socio-economic score, height, and alcohol consumption were included in the model as continuous terms; all other predictors were categorical. Age was mean-corrected (i.e., the reference value was the mean) and height was used as sex-specific standardised score (See Equation (1) below) due to the bimodal distribution of height by sex. We included the squared term for age (in addition to age as a continuous term) to capture non-linear effects. We categorised ethnicity according to ancestral risk of melanoma, as follows: 'high risk' (British/European ancestry), moderate risk (Middle Eastern or South American ancestry), and low risk (Aboriginal or Torres Strait Island, South Sea Islander, Asian, or other ancestry).

$$height\ score_i = \frac{height_i - \mu_{sex\,(i)}}{\sigma_{sex\,(i)}} \qquad (1)$$

Where:

$height_i$ - Observed height of ith individual
$\mu_{sex\,(i)}$ - Mean height for the sex of ith individual (eg. Male or Female)
$\sigma_{sex\,(i)}$ - Standard deviation of height for the sex of ith individual

Imputation of Missing data

The prevalence of missing values for most candidate predictors was low (<3%), but ranged up to 15% for family history of melanoma (Table 1, Supplementary Table 1). To minimize potential bias due to missing data, we performed multivariate imputation by chained equations (MICE) assuming data were missing completely at random. The MICE model included all potential predictors, as well survival time, outcome status, and the Nelson-Aalen hazard estimate. Based on the overall percentage missing, we created 50 multiply imputed datasets.

Outcomes

© 2025 Whiteman DC et al. *JAMA Dermatology*.

The primary outcome was first histological diagnosis of invasive melanoma.

Sample size
We followed the sample size guideline for developing clinical prediction models.[4] Briefly, using the full list of candidate predictors, we estimated the Nagalkerke R^2 to be 0.0464. Assuming ~10% shrinkage during model development, we estimated about 22 events would be required per predictor parameter for optimum model development and performance. With about 700 events in the cohort over 10 years of follow-up, our calculations suggested a model with 33 parameters would be optimum. As detailed above, we pre-specified a list of 31 predictor variables (comprising 27 phenotypic/clinical variables and four statistical interaction variables) based on previously identified risk factors, as well as potential predictors of clinical significance from the literature. Fitting these 31 predictor variables required a set of 64 separate parameters. Rather than arbitrarily selecting one candidate predictor variable from among several correlated variables (e.g. sunburns as a child vs sunburns as a teenager/youth vs sunburns as an adult), we let the variable selection process select from among the candidate predictors based on minimum Akaike Information Criteria (AIC; see below).

Statistical analysis
We used Cox proportional hazards approaches to derive a risk prediction model for the onset of newly diagnosed invasive melanoma. We followed the TRIPOD (Transparent Reporting of a multivariable prediction model for Individual Prognosis or Diagnosis) checklist for model development and performance assessment.[5] We used all available data to develop the model, and used resampling methods for internal validation.
Follow-up time for statistical analysis commenced at the date of study entry (hereafter 'baseline') and continued until a maximum of 10 years duration. Follow-up time ceased on the date of the first histologically confirmed diagnosis of invasive cutaneous melanoma, or at 10 years follow-up, whichever occurred first. We censored participants early on the date of their death or the date of diagnosis of *in situ* melanoma, if those events occurred before the primary outcome or administrative censoring date.
We applied a stepwise approach to variable selection using the full set of potential predictors. We used the minimum AIC method and included both forward selection and backward elimination processes. We executed variable selection for each of the 50 imputed datasets. We pre-specified that predictors selected in at least 35 of 50 imputed datasets (70%) were retained in the final risk prediction model. To estimate the apparent effect estimates, we pooled effect estimates from the final model from each of the 50 imputed datasets using Rubin's rules.[6] Cumulative hazards were log transformed before pooling and the pooled value was then exponentiated. We assessed the proportional hazard assumptions for the predictors included in the final developed model in all 50 imputed datasets individually.

Model performance and internal validation
We assessed model performance using discrimination and calibration. We calculated the Concordance index (C-index) and Brier score (mean squared error of predictions) for the final developed risk prediction model in each imputed dataset and reported the pooled estimates of these performance metrics.
For internal validation and calibration, we generated 1000 bootstrap samples from one of the multiply imputed datasets. We used the full list of candidate predictors and stepwise selection with minimum AIC criteria for validation and calibration for bootstrap samples, as recommended[7] We used DFBETA residuals (a measure of influence of each observation on the regression coefficient) to estimate uniform shrinkage and parameter-wise shrinkage.[8] For parameter-wise shrinkage we used the joint shrinkage factor for each categorical predictor. We present bias-corrected parameter estimates using parameter-wise joint shrinkage.
To assess the stability of effect estimates, we plotted estimated risk at 10 years based on the final developed model using one imputed dataset against the same estimates from the 1000 bootstrap sample models. We estimated mean absolute prediction error (MAPE) for each individual as an average of the absolute difference between the risk predicted by the developed risk prediction model and each of the 1000 bootstrap models. Overall MAPE was estimated as the average MAPE for the whole cohort.

Clinical utility
To inform decisions about how best to stratify the population based on melanoma risk, we grouped participants into decile risk groups based on their individual risk scores from the developed model. For each risk group, we then estimated their cumulative hazard at 10 years and plotted it against their observed risk. We then calculated the Youden index [9] for each decile as a means of assessing the threshold at which both sensitivity and specificity were optimised. We performed decision curve analysis (DCA) to assess the net benefit of using model for screening at different thresholds compared to screening all and screening none. [10]
Lastly, we compared the performance of the new tool against our previously developed 7-item prediction tool[11] by comparing sensitivities of the two models at decile cut-points to 10 years of follow-up, and then calculating the net reclassification index (NRI) under different thresholds for targeted screening. [12]

© 2025 Whiteman DC et al. *JAMA Dermatology*.

Sensitivity analysis

To examine the influence of censoring participants diagnosed with melanomas *in situ* during follow-up, we conducted two sensitivity analyses: (1) we assessed the performance of the derived model using the data which ignored all diagnoses of melanoma *in situ*; and (2) we separately re-ran the entire model derivation process ignoring all diagnoses of melanoma *in situ* (i.e. using forwards and backwards model selection in all 50 imputed datasets).

© 2025 Whiteman DC et al. *JAMA Dermatology*.

References

1. Olsen CM, Green AC, Neale RE, Webb PM, Cicero RA, Jackman LM, O'Brien SM, Perry SL, Ranieri BA, Whiteman DC, Study QS. Cohort profile: the QSkin Sun and Health Study. *Int J Epidemiol* 2012; **41**(4): 929-i.

2. Morze CJ, Olsen CM, Perry SL, Jackman LM, Ranieri BA, O'Brien SM, Cicero RA, Whiteman DC, Study QS. Good test-retest reproducibility for an instrument to capture self-reported melanoma risk factors. *J Clin Epidemiol* 2012; **65**(12): 1329-36.

3. Mortimore A, Pandeya N, Olsen CM, Whiteman DC. "Repeatability of Repeatability": the stability of self-reported melanoma risk factors in two independent samples. *Aust N Z J Public Health* 2021; **45**(5): 469-73.

4. Riley RD, Ensor J, Snell KIE, Harrell FE, Jr., Martin GP, Reitsma JB, Moons KGM, Collins G, van Smeden M. Calculating the sample size required for developing a clinical prediction model. *BMJ* 2020; **368**: m441.

5. Collins GS, Reitsma JB, Altman DG, Moons KG. Transparent reporting of a multivariable prediction model for individual prognosis or diagnosis (TRIPOD): the TRIPOD statement. *BMJ* 2015; **350**: g7594.

6. Rubin DB, Schafer JL. Efficiently creating multiple imputations for incomplete multivariate normal data. Proceedings of the Statistical Computing Section of the American Statistical Association; 1990: American Statistical Association Alexandria, VA, USA; 1990. p. 88.

7. Efthimiou O, Seo M, Chalkou K, Debray T, Egger M, Salanti G. Developing clinical prediction models: a step-by-step guide. *BMJ* 2024; **386**: e078276.

8. Dunkler D, Sauerbrei W, Heinze G. Global, parameterwise and joint shrinkage factor estimation. *Journal of Statistical Software* 2016; **69**: 1-19.

9. Youden WJ. Index for rating diagnostic tests. *Cancer* 1950; **3**(1): 32-5.

10. Vickers AJ, Elkin EB. Decision curve analysis: a novel method for evaluating prediction models. *Med Decis Making* 2006; **26**(6): 565-74.

11. Olsen CM, Pandeya N, Thompson BS, Dusingize JC, Webb PM, Green AC, Neale RE, Whiteman DC, Study QS. Risk Stratification for Melanoma: Models Derived and Validated in a Purpose-Designed Prospective Cohort. *J Natl Cancer Inst* 2018; **110**(10): 1075-83.

12. Kerr KF, Wang Z, Janes H, McClelland RL, Psaty BM, Pepe MS. Net reclassification indices for evaluating risk prediction instruments: a critical review. *Epidemiology* 2014; **25**(1): 114-21.

© 2025 Whiteman DC et al. *JAMA Dermatology*.

eTable 1. Distribution of demographic, socio-economic and phenotypic characteristics that were not retained in the final model: overall for the QSkin cohort (n=41,919) and according to incident invasive melanoma status.

Characteristic	Overall (n = 41,919)	No melanoma (n = 41,213)	Invasive Melanoma (n = 706)	p-value[1]
	N (%)	N (%)	N (%)	
**Index of socioeconomic disadvantage**				0.6
Median (IQR)	1,006.1 (954.3-1,040.4)	1,005.8 (954.3-1,040.4)	1,006.8 (958.7-1,039.7)	
Missing	*113*	*107*	*6*	
**Accessibility/Remoteness index of Australia**				0.7
Major cities	25,106 (60)	24,678 (60)	428 (61)	
Inner regional	10,134 (24)	9,961 (24)	173 (25)	
Outer/remote/rural	6,639 (16)	6,535 (16)	104 (15)	
Missing	*40*	*39*	*1*	
**Age migrated to Australia**				<0.001
Native born	33,514 (80)	32,909 (80)	605 (86)	
<15 years	2,651 (6.4)	2,611 (6.4)	40 (5.7)	
15 years or more	5,572 (13)	5,511 (13)	61 (8.6)	
Missing	*182*	*182*	*0*	
**Region lived longest before age 20**				<0.001
Lived overseas	4,735 (12)	4,686 (12)	49 (7.2)	
Southern Australia	5,968 (15)	5,870 (15)	98 (14)	
Central Australia	17,397 (43)	17,073 (43)	324 (47)	
Northern Australia	12,099 (30)	11,887 (30)	212 (31)	
Missing	*1,720*	*1,697*	*23*	
**Work status**				<0.001
Full time	19,402 (47)	19,101 (47)	301 (44)	
Part time	7,418 (18)	7,311 (18)	107 (15)	
Retired	9,155 (22)	8,940 (22)	215 (31)	
Other	5,007 (12)	4,939 (12)	68 (9.8)	
Missing	*937*	*922*	*15*	
**Burning tendency**				<0.001
Not burn	3,942 (9.5)	3,906 (9.5)	36 (5.1)	
Burn a little	18,186 (44)	17,928 (44)	258 (37)	
Burn moderately	13,833 (33)	13,573 (33)	260 (37)	
Burn badly	5,699 (14)	5,552 (14)	147 (21)	
Missing	*259*	*254*	*5*	
**Eye colour**				0.002
Blue/grey	15,827 (38)	15,536 (38)	291 (42)	
Green/hazel	15,456 (37)	15,184 (37)	272 (39)	
Brown/black	10,049 (24)	9,918 (24)	131 (19)	
Missing	*587*	*575*	*12*	
**Sunburns as a child**				<0.001
Never	8,392 (22)	8,284 (22%)	108 (17)	
1-10	23,572 (62)	23,165 (62%)	407 (64)	
11-20	3,563 (9.4)	3,499 (9.4%)	64 (10)	
>20	2,292 (6.1)	2,235 (6.0%)	57 (9.0)	
Missing	*4,100*	*4,030*	*70*	
**Sunburns as a youth**				<0.001
Never	2,260 (5.6)	2,248 (5.7)	12 (1.7)	
1-10	27,472 (68)	27,021 (68)	451 (66)	
11-20	6,620 (16)	6,489 (16)	131 (19)	

© 2025 Whiteman DC et al. *JAMA Dermatology*.

Characteristic	**Overall** (n = 41,919)	**No melanoma** (n = 41,213)	**Invasive Melanoma** (n = 706)	**p-value**[1]
	N (%)	N (%)	N (%)	
>20	4,004 (9.9)	3,912 (9.9)	92 (13)	
Missing	*1,563*	*1,543*	*20*	
**Sunbed use**				0.4
Never	37,244 (89)	36,609 (89)	635 (90)	
Once or more	4,379 (11)	4,312 (11)	67 (9.5)	
Missing	*296*	*292*	*4*	
**Number of alcoholic drinks per week**[2]				<0.001
0	8,121 (19)	8,012 (20)	109 (16)	
0.5	6,981 (17)	6,855 (17)	126 (18)	
3	7,582 (18)	7,480 (18)	102 (15)	
5.5	5,446 (13)	5,350 (13)	96 (14)	
10	6,563 (16)	6,428 (16)	135 (19)	
17	3,560 (8.5)	3,478 (8.5)	82 (12)	
24	1,641 (3.9)	1,618 (3.9)	23 (3.3)	
30	1,797 (4.3)	1,768 (4.3)	29 (4.1)	
Missing	*228*	*224*	*4*	

[1] Pearson's Chi-squared test for categories and Wilcoxon rank sum test for continuous predictors.
[2] Number of drinks was used as an ordinal variable in the model

© 2025 Whiteman DC et al. *JAMA Dermatology*.

eTable 2. Tumour characteristics of the invasive melanomas included in the risk prediction model (n=706).

Tumour characteristics	Number of cases (%)
**Melanoma type**	
Superficial spreading	408 (57.8)
Nodular	51 (7.2)
Lentigo maligna	57 (8.1)
Acral lentiginous	2 (0.3)
Other (not specified)	188 (26.6)
**Site**	
Head/Neck	106 (15.0)
Trunk	258 (36.5)
Upper limbs	196 (27.8)
Lower limbs	137 (19.4)
Missing	*9*
**Thickness**	
<1 mm	532 (77.3)
1-1.99 mm	94 (13.7)
2-3.99 mm	37 (5.4)
>=4 mm	25 (3.6)
Missing	*18*

© 2025 Whiteman DC et al. *JAMA Dermatology*.

eTable 3. Frequency of selection of candidate predictors from 50 imputed datasets during model derivation using minimum AIC selection criteria.

List of potential factors for the risk prediction model	Number of times selected out of 50 imputed datasets
Age at baseline	50
Square term of age at baseline	50
Sex	50
Index of socioeconomic disadvantage	0
Accessibility/Remoteness index of Australia	0
Age migrated to Australia	0
Region lived longest before age 20	0
Education level	0
Work status	0
Burning tendency	0
Tanning ability	50
Eye colour	0
Hair colour	50
Freckles at age 21	50
Moles at age 21	50
Sunburns as a child	12
Sunburns as a youth	2
Sunburns as an adult	50
Sunbed use	1
Skin cancer excisions prior to baseline	50
Sunspots treated prior to baseline	50
Family history of melanoma	41
Height	50
Number of alcoholic drinks per week	0
Baseline smoking status	50
Other cancer prior to baseline	50
Ancestral risk of melanoma	50
Interaction: Age by Sex	50
Interaction: Age by Moles at age 21	0
Interaction: Age by Skin cancer excisions prior to baseline	0
Interaction: Age Sunspots treated prior to baseline	0

© 2025 Whiteman DC et al. *JAMA Dermatology*.

eTable 4. Assessment of the proportional hazards assumption for the 16 predictors retained in the final model.

Variables	Chi-square	df	P values
Age at baseline	0.2985	1	0.58
Square term of age at baseline	4.2045	1	0.04
Sex	0.0984	1	0.75
Ancestral risk of melanoma	0.6962	1	0.40
Other cancer prior to baseline	0.2240	1	0.64
Tanning ability	4.3109	3	0.23
Hair colour	1.5948	2	0.45
Freckles at age 21	5.1816	3	0.16
Moles at age 21	2.240	3	0.53
Sunburns as an adult	5.7768	2	0.12
Skin cancer excisions prior to baseline	0.3290	2	0.85
Sunspots treated prior to baseline	1.2764	3	0.73
Family history of melanoma	0.3708	1	0.54
Baseline smoking status	0.0301	2	0.99
Standardised score for height	2.6888	1	0.10
Age by sex interaction	0.0260	1	0.87
Global assessment	33.3352	31	0.31

© 2025 Whiteman DC et al. *JAMA Dermatology*.

eTable 5. Frequency of selection of candidate predictors during model derivation in 1000 bootstrapped datasets, in descending order of frequency.

	Predictors	Frequency of selection	Variable inclusion frequency (%)
1	Moles at age 21	1000	100
2	Sex	994	99.4
3	Tanning ability	950	95.0
4	Sunspots treated prior to baseline	976	97.6
5	Skin cancer excisions prior to baseline	902	90.2
6	Other cancer prior to baseline	865	86.5
7	Freckles at age 21	798	79.8
8	Hair colour	670	67.0
9	Ancestral risk of melanoma	557	55.7
10	Age at baseline	506	50.6
11	Interaction: Age by Sex	426	42.6
12	Sunburns as an adult	383	8.3
13	Baseline smoking status	318	31.8
14	Height score	243	24.3
15	Square term of age at baseline	236	23.6
17	Family history of melanoma	177	17.7
18	Sunburns as a youth	134	13.4
19	Sunburns as a child	132	13.2
20	Interaction: Age by Sunspots treated prior to baseline	105	10.5
21	Interaction: Age by Moles at age 21	88	<1
22	Sunbed use	74	<1
23	Work status	74	<1
24	Region lived longest before age 20	66	<1
25	Education level	61	<1
26	Burning tendency	60	<1
27	Index of socioeconomic disadvantage	40	<1
28	Eye colour	32	<1
29	Age migrated to Australia	19	<1
30	Accessibility/Remoteness index of Australia	15	<1
31	Number of alcoholic drinks per week	16	<1
32	Interaction: Age by Skin cancers excised prior to baseline	11	<1

© 2025 Whiteman DC et al. *JAMA Dermatology*.

eTable 6. Parameter-wise shrinkage and bias-corrected beta estimates (pooled estimates of 50 multiply imputed datasets).

Predictors	Pooled parameter-wise shrinkage[1] (SE)	Bias-corrected beta estimates (SE)
**Age**	0.94 (0.33)	0.022 (0.01)
**Age squared**	0.77 (0.44)	-0.001 (0.001)
**Sex**		
Female	Reference	Reference
Male	0.95 (0.17)	0.47 (0.08)
**Ancestral risk of melanoma**		
Moderate/low risk	Reference	Reference
High risk	0.93 (0.31)	1.08 (0.36)
**Moles at age 21**		
None	Reference	Reference
A few	0.97 (0.12)	0.30 (0.10)
Some	0.97 (0.12)	0.82 (0.11)
Many	0.97 (0.12)	1.07 (0.18)
**Hair colour**		
Black	Reference	Reference
Dark/light brown/blonde	0.91 (0.26)	0.42 (0.16)
Red/auburn	0.91 (0.26)	0.70 (0.19)
**Freckles at age 21**		
None	Reference	Reference
A few	0.89 (0.24)	0.18 (0.09)
Some	0.89 (0.24)	0.39 (0.10)
Many	0.89 (0.24)	0.21 (0.14)
**Tanning ability**		
Deeply	Reference	Reference
Tan moderately	0.94 (0.20)	0.29 (0.11)
Tan lightly	0.94 (0.20)	0.49 (0.13)
Not tan	0.94 (0.20)	0.70 (0.16)
**Sunburns as an adult**		
None	Reference	Reference
1-10	0.71 (0.34)	0.220 (0.09)
11-20	0.71 (0.34)	0.11 (0.12)
>20	0.71 (0.34)	0.34 (0.13)
**Family history of melanoma**		
No	Reference	Reference
Yes	0.67 (0.63)	0.11 (0.08)
**Other cancer prior to baseline**		
No	Reference	Reference
Yes	0.94 (0.24)	0.45 (0.11)
**Baseline smoking status**		
Never smoker	Reference	Reference
Past smoker	0.74 (0.34)	-0.17 (0.11)
Current smoker	0.74 (0.34)	-0.17 (0.06)
**Skin cancer excisions prior to baseline**		
None	Reference	Reference
One	1.01 (0.23)	0.22 (0.12)
More than one	1.01 (0.23)	0.44 (0.10)
**Sunspots treated prior to baseline**		
None	Reference	Reference

© 2025 Whiteman DC et al. *JAMA Dermatology*.

Predictors	Pooled parameter-wise shrinkage[1] (SE)	Bias-corrected beta estimates (SE)
1-5	0.99 (0.23)	0.46 (0.11)
6-20	0.99 (0.23)	0.50 (0.13)
>20	0.99 (0.23)	0.66 (0.14)
**Height (per one SD)**	0. 80 (0.45)	0.07 (0.03)
**Age by sex interaction**	0.91 (0.39)	0.02 (0.01)

[1] Parameter-wise shrinkage was estimated based on DFBETA residuals with joint shrinkage for multiple levels of categorical predictors using the R package shrink. SE-standard error.

© 2025 Whiteman DC et al. *JAMA Dermatology*.

eTable 7. Case distribution in risk deciles based on the previous QSkin risk prediction model and the current model for invasive melanoma after 10 years follow up.

Decile group[1]	MP7[2]				MP16[3]			
	True positive	Sensitivity (%)	Specificity (%)	Cumulative number needed to screen	True positive	Sensitivity (%)	Specificity (%)	Cumulative number needed to screen
10	204	28.9	90.3	21	230	32.6	90.4	18
9	114	45.0	80.6	26	112	48.4	80.5	25
8	103	59.6	70.5	30	107	63.6	70.6	28
7	71	69.7	60.5	34	76	74.4	60.6	32
6	53	77.2	50.5	38	55	82.2	50.6	36
5	60	85.7	40.7	41	53	89.7	40.5	40
4	38	91.1	30.4	46	33	94.3	30.4	44
3	27	94.9	20.3	50	20	97.2	20.3	49
2	27	98.7	10.2	54	13	99.0	10.2	54
1	9	100.0	0.0	59	7	100.0	0.0	59

[1]Highest risk decile listed first
[2]Previously published model with a list of 7 predictors
[3]Current model with a list of 16 predictors

© 2025 Whiteman DC et al. *JAMA Dermatology*.

eTable 8. Results of sensitivity analyses ignoring incident melanoma *in situ* (pooled estimates of 50 multiply imputed datasets).

Predictors	Sensitivity analysis 1[1] Pooled Hazard Ratio (95%CI)	Sensitivity analysis 2[2] Pooled Hazard Ratio (95%CI)
**Age**	1.02 (1.01-1.04)	1.02 (1.01-1.04)
**Age squared**	1.00 (1.00-1.00)	1.00 (1.00-1.00)
**Sex**		
Female	Reference	Reference
Male	1.61 (1.37-1.89)	1.65 (1.40-1.94)
**Ancestral risk of melanoma**		
Moderate/low risk	Reference	Reference
High risk	3.01 (1.49-6.08)	3.00 (1.48-6.05)
**Moles at age 21**		
None	Reference	Reference
A Few	1.39 (1.15-1.68)	1.39 (1.15-1.68)
Some	2.28 (1.83-2.84)	2.28 (1.83-2.85)
Many	3.33 (2.39-4.65)	3.34 (2.39-4.65)
**Hair colour**		
Black	Reference	Reference
Dark/light brown/blonde	1.42 (1.04-1.93)	1.41 (1.04-1.92)
Red/auburn	2.02 (1.38-2.97)	2.02 (1.37-2.96)
**Freckles at age 21**		
None		
A few	1.20 (1.00-1.43)	1.19 (1.00-1.43)
Some	1.51 (1.23-1.86)	1.51 (1.23-1.85)
Many	1.28 (0.95-1.73)	1.28 (0.95-1.73)
**Tanning ability**		
Tan deeply	Reference	Reference
Tan moderately	1.40 (1.11-1.76)	1.41 (1.12-1.77)
Tan lightly	1.72 (1.33-2.21)	1.73 (1.34-2.23)
Not tan	2.00 (1.46-2.75)	2.03 (1.48-2.78)
**Sunburns as an adult**		
None	Reference	Reference
1-10	1.32 (1.05-1.66)	1.31 (1.04-1.65)
11-20	1.23 (0.89-1.69)	1.21 (0.88-1.66)
>20	1.58 (1.12-2.18)	1.55 (1.10-2.18)
**Family history of melanoma**		
No	Reference	Reference
Yes	1.16 (0.98-1.37)	1.16 (0.98-1.38)
**Other cancer prior to baseline**		
No	Reference	Reference
Yes	1.56 (1.26-1.93)	1.56 (1.26-1.93)
**Baseline smoking status**		
Never smoker	Reference	Reference
Past smoker	0.81 (0.69-0.95)	0.81 (0.69-0.95)
Current smoker	0.86 (0.65-1.13)	0.86 (0.65-1.13)
**Skin cancer excisions prior to baseline**		
None	Reference	Reference
One	1.25 (0.99-1.57)	1.25 (0.99-1.57)
More than one	1.57 (1.29-1.90)	1.57 (1.29-1.90)
**Sunspots treated prior to baseline**		
None	Reference	Reference

© 2025 Whiteman DC et al. *JAMA Dermatology*.

Predictors	Sensitivity analysis 1[1] Pooled Hazard Ratio (95%CI)	Sensitivity analysis 2[2] Pooled Hazard Ratio (95%CI)
1-5	1.56 (1.25-1.93)	1.56 (1.25-1.93)
6-20	1.68 (1.32-2.13)	1.68 (1.32-2.13)
>20	2.00 (1.54-2.60)	2.00 (1.54-2.60)
**Sunbed use**		
Ever		Reference
Never		1.23 (0.96-1.58)
**Height (per one SD)**	1.09 (1.01-1.17)	1.09 (1.01-1.17)
**Age by sex interaction**	1.03 (1.01-1.05)	1.03 (1.01-1.05)
**Pooled C-index**	0.74 (0.73-0.76)	0.74 (0.73-0.76)

[1] Hazard ratios for the predictors in the final risk prediction model when incident melanoma *in situ* were ignored.
[2] Hazard ratios for the predictors in the final risk prediction model after re-running the variable selection step ignoring incident melanoma *in situ*.

© 2025 Whiteman DC et al. *JAMA Dermatology*.

eFigure 1. Survival probability for the outcome (invasive melanoma) and censoring events (melanoma *in situ*, death) over 10 years of follow-up.

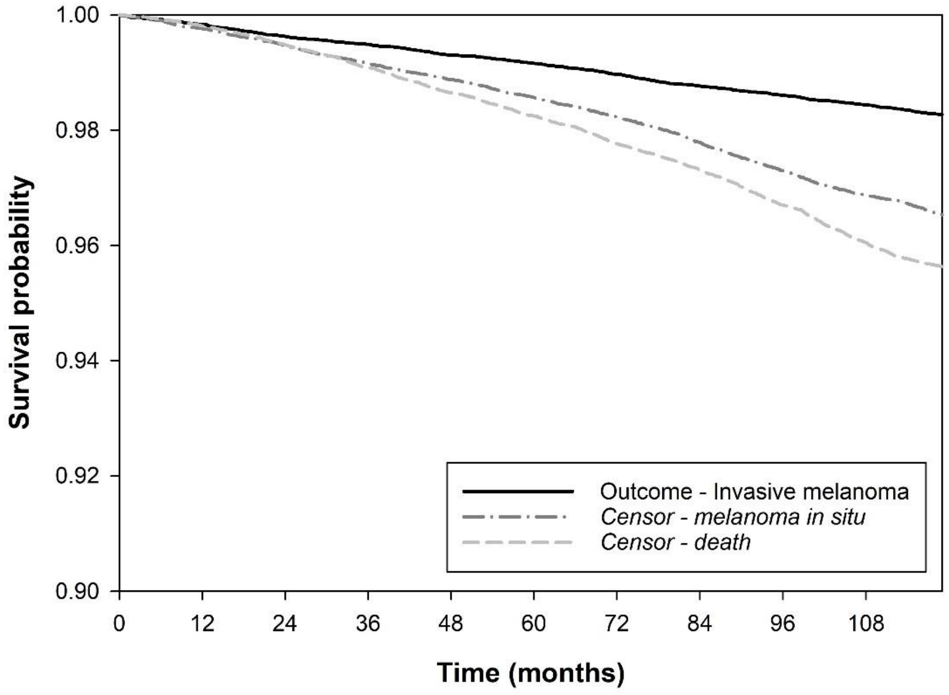

© 2025 Whiteman DC et al. *JAMA Dermatology*.

eFigure 2. Time dependent C-index* for the developed model. (*single imputed dataset)

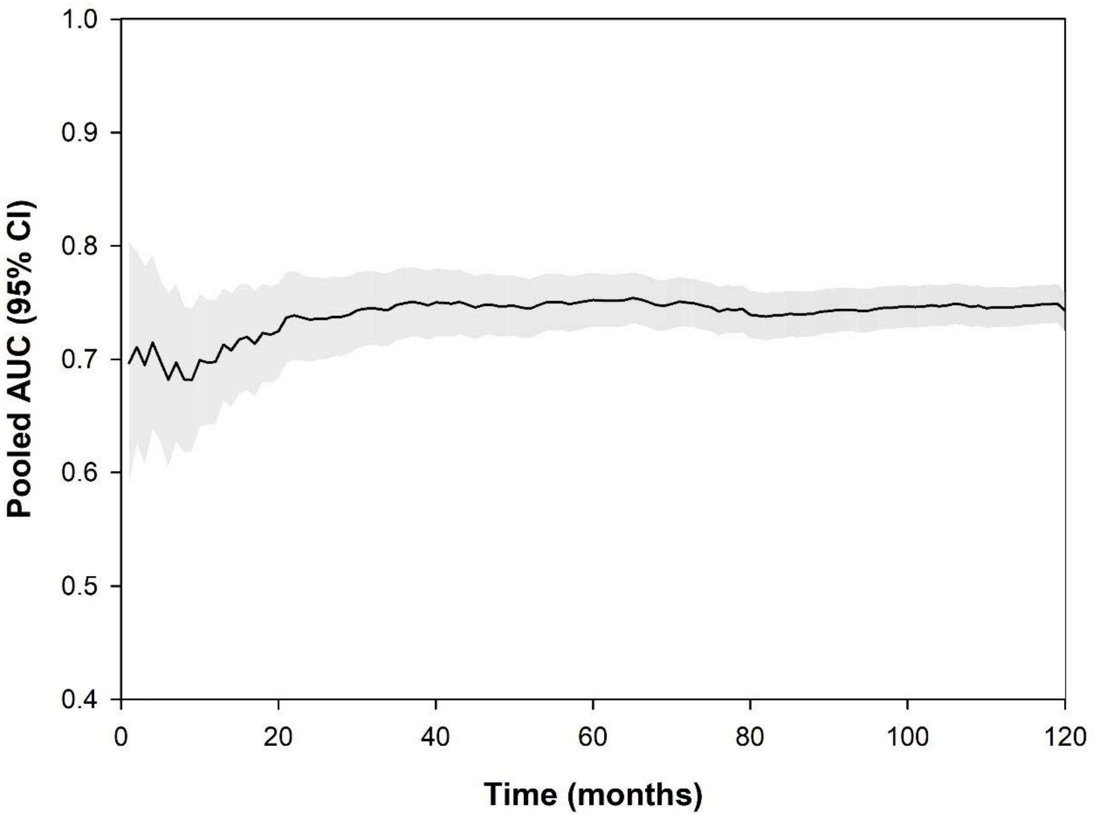

© 2025 Whiteman DC et al. *JAMA Dermatology*.

eFigure 3. Observed versus predicted risk* for: a) the developed model; and b) the 1000 bootstrap sample. (*single imputed dataset)

a)

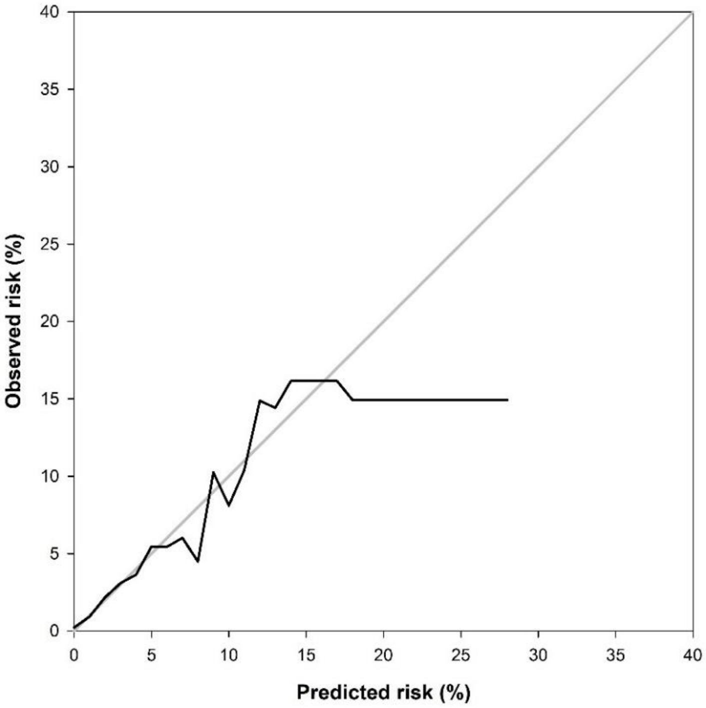

b)

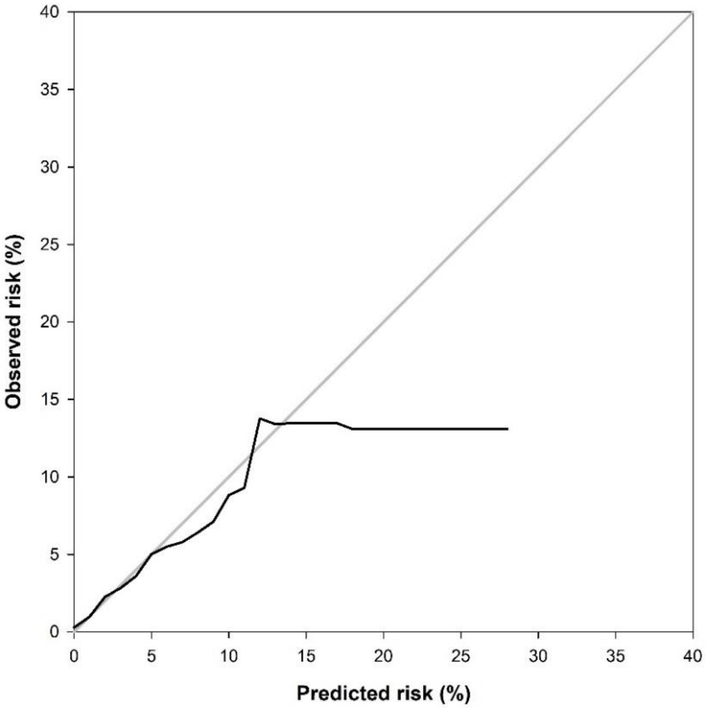

© 2025 Whiteman DC et al. *JAMA Dermatology*.

eFigure 4. Area under the curve* for the 1000 bootstrap sample. (*single imputed dataset)

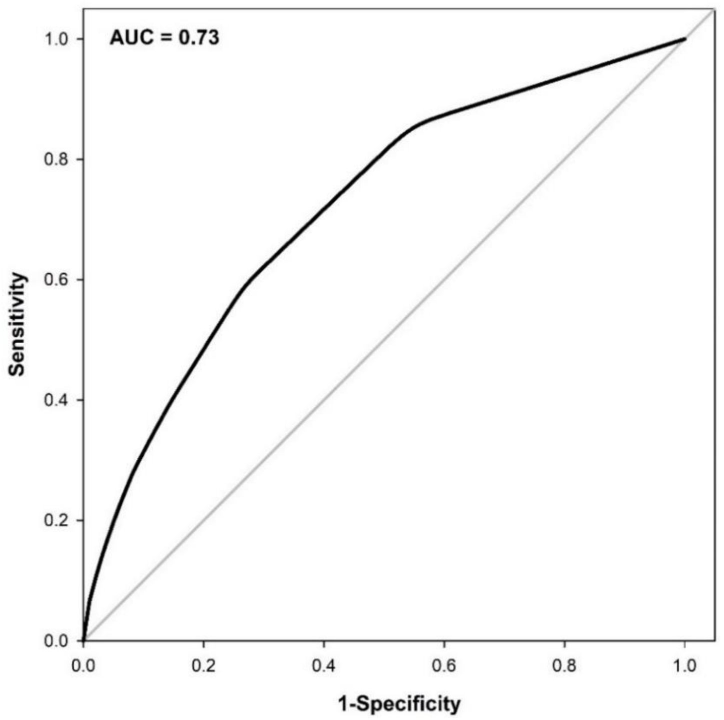

© 2025 Whiteman DC et al. *JAMA Dermatology*.

eFigure 5. Mean absolute prediction error and estimated risk at 10 year using final risk prediction model for each individual. Mean absolute prediction error is the average of the difference in predicted risk from developed model to that in the bootstrap sample averaged over 1000 bootstraps for each individual. Each dot refers to an individual.

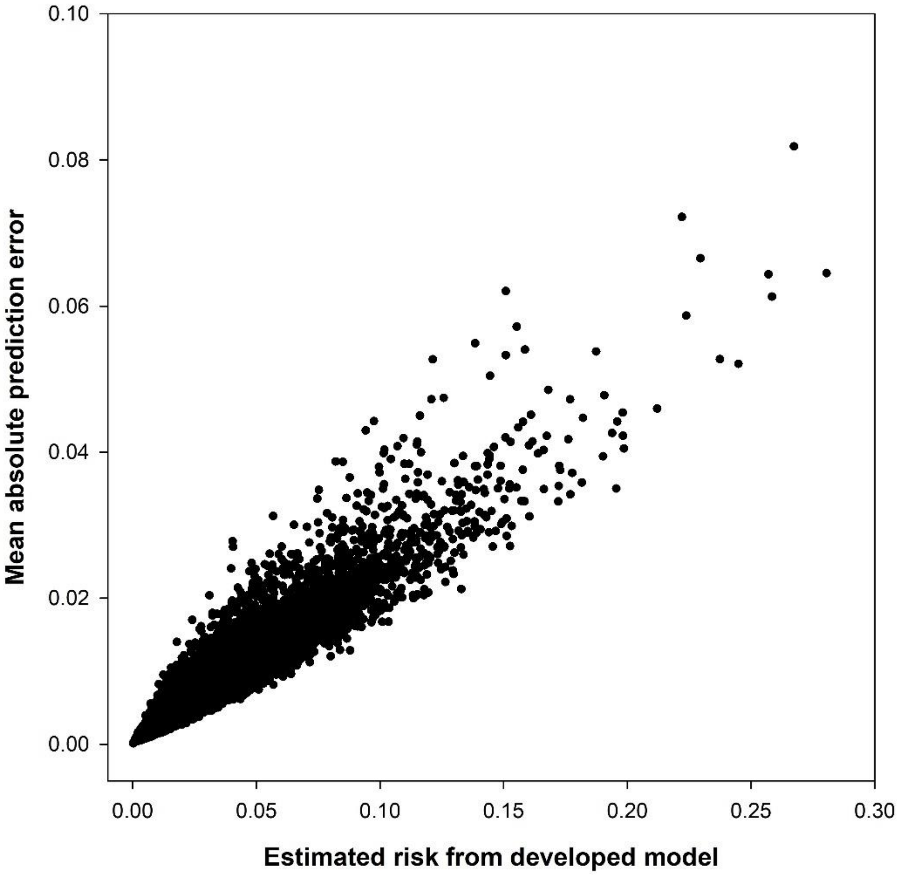

© 2025 Whiteman DC et al. *JAMA Dermatology*.

eFigure 6. Decision curve analysis plotting net benefit against the threshold probability

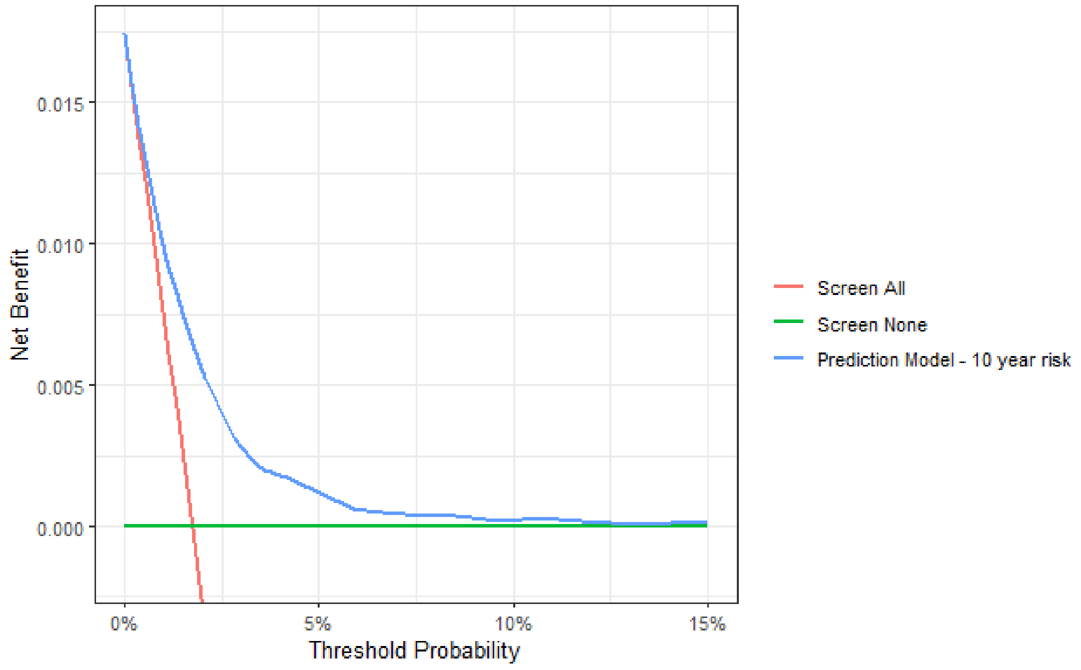

© 2025 Whiteman DC et al. *JAMA Dermatology*.
