# Supplementary material for: A Risk Prediction Tool for Invasive Melanoma
Source: JAMA Dermatol. 2025 Sep 10;161(11):1123–31. doi: 10.1001/jamadermatol.2025.3028 (PMC12423951; doi:10.1001/jamadermatol.2025.3028)
Supplement: Supplement 2. — Data sharing statement [file jamadermatol-e253028-s002.pdf]

Data Sharing Statement

Whiteman. A Risk Prediction Tool for Invasive Melanoma. *JAMA Dermatol*. Published September 10, 2025. doi:10.1001/jamadermatol.2025.3028

Data
Data available: Yes
Data types: Other (please specify)
Additional Information: The authors will make the de-identified, relevant data from these analyses available to requestors, subject to approval from the requestor's institutional ethics committee and the human research ethics committee of QIMR Berghofer Medical Research Institute. Codes used for the analyses may be available on request. The model will be available at the QIMR Berghofer website for general access (www.qimrb.edu.au/melanoma).
How to access data: David.Whiteman@qimrberghofer.edu.au
When available: With publication

Supporting Documents
Document types: None

Additional Information
Who can access the data: As per the data sharing statement provided above.
Types of analyses: Subject to discussion with PI and regulatory clearances.
Mechanisms of data availability: Subject to discussion with PI and regulatory clearances.
